# Excision and suture in the midline *versus* Karydakis flap surgery for pilonidal sinus: randomized clinical trial

**DOI:** 10.1093/bjsopen/zrac007

**Published:** 2022-03-15

**Authors:** Oskar Hemmingsson, Felix Binnermark, Christoffer Odensten, Martin Rutegård, Karl A. Franklin, Markku M. Haapamäki

**Affiliations:** 1 Department of Surgical and Perioperative Sciences, Surgery, Umeå University, Umeå, Sweden; 2 Wallenberg Centre for Molecular Medicine, Umeå University, Umeå, Sweden; 3 Department of Surgical and Perioperative Sciences, Surgery, Umeå University Educational Unit at Sunderby Hospital, Sunderby, Sweden

## Abstract

**Background:**

There are several surgical options for the management of pilonidal disease, including midline and off midline closure, but prospective studies are rare. The study hypothesis was that Karydakis flap surgery would result in shorter wound healing and fewer recurrences than excision of pilonidal sinus and suture in the midline.

**Methods:**

A randomized clinical trial was conducted in two hospitals in Sweden between 2006 and 2015 to compare excision and suture in the midline with Karydakis flap surgery. Adult patients with a chronic pilonidal sinus disease were randomized 1:1 at the outpatient clinic without blinding. Power calculation based on recurrence of 2 per cent for Karydakis flap and 10 per cent for excision and primary closure in the midline required 400 patients with 90 per cent statistical power at 5 per cent significance assuming 10 per cent loss during follow-up. Participants were followed up until complete wound healing; late follow-up after 6–13 years was performed by telephone by two blinded assessors. The two co-primary outcomes were time to complete wound healing and recurrence rate.

**Results:**

The study was terminated early at a planned interim analysis due slow recruitment and a significant difference in primary outcome. In total, 125 patients were randomized, of whom 116 were available for the present analysis. Median wound healing time was 49 days (95 per cent confidence interval (c.i.) 32 to 66) for excision with suture in the midline and 14 days (95 per cent c.i. 12 to 20) for Karydakis flap surgery (*P* < 0.001). There were five recurrences in each group, after a median follow-up of 11 years (*P* = 0.753).

**Conclusion:**

Karydakis flap surgery for pilonidal sinus disease led to a shorter wound healing time than excision and suture in the midline but no difference in recurrence rates.

Registration number: NCT00412659 (http://www.clinicaltrials.gov)

## Teaser

Two surgical techniques for pilonidal sinus disease were studied in an expertise-based randomized controlled trial with long term follow-up. Karydakis flap surgery for pilonidal sinus disease leads to a shorter wound healing time than excision and suture in the midline, while the recurrence rate is similar.

## Introduction

Chronic pilonidal sinus disease mostly affects young adults and is more common in men^1,2^. Without effective treatment, it causes a painful lump or secretion of pus through one or several skin openings in the intergluteal area; the condition can persist for years^3^.

Several surgical techniques are used to treat pilonidal sinus disease, but there is a lack of consensus regarding best surgical practice^4,5^. Meta-analyses indicate advantages for flap techniques^6–8^ such as the Karydakis flap procedure, which is an advancement flap known to be one of the simplest off-midline procedures^9^. Despite this, in many countries, the most common technique is excision of the pilonidal sinus and tension-free primary suture in the midline^10^. According to one comparative study^11^, the Karydakis technique had fewer complications with fewer recurrences than midline closure. The only randomized controlled trial (RCT) to compare these techniques reported no significant differences in postoperative complication and recurrence rates after Karydakis surgery compared with primary excision and tension-free midline closure, while wound healing was not reported^12^. The present RCT was conducted to test if Karydakis flap surgery results in shorter wound healing time and fewer recurrences than excision and tension-free primary suture in the midline.

## Methods

### Study design

This was a prospective, pragmatic, expertise-based, randomized controlled clinical trial.

### Participants

Inclusion criteria were adult patients aged 18 years of age or older with primary or once-recurrent pilonidal disease considered for surgical treatment. Patients were included if at least 4 weeks had elapsed since any acute incision for a pilonidal abscess.

Patients considered for only minimally invasive procedures (shaving, picking loose hair from skin openings, or minimal curettage) were excluded. Patients were informed and asked for consent to participate by the attending surgeon who assigned participants at the outpatient clinic. Approval from the Regional Ethical Review Board of Umeå University was obtained in January 2006 (registration number 05-178M).

### Interventions

**Fig. 1:**
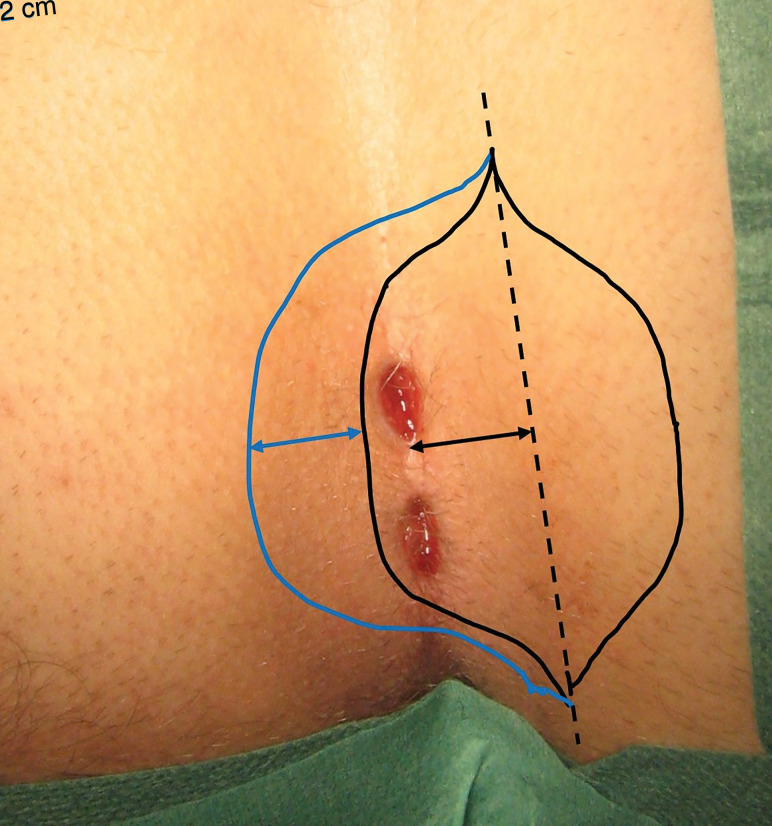
Skin incision in Karydakis flap surgery is marked with a black line on the photograph

**Fig. 2:**
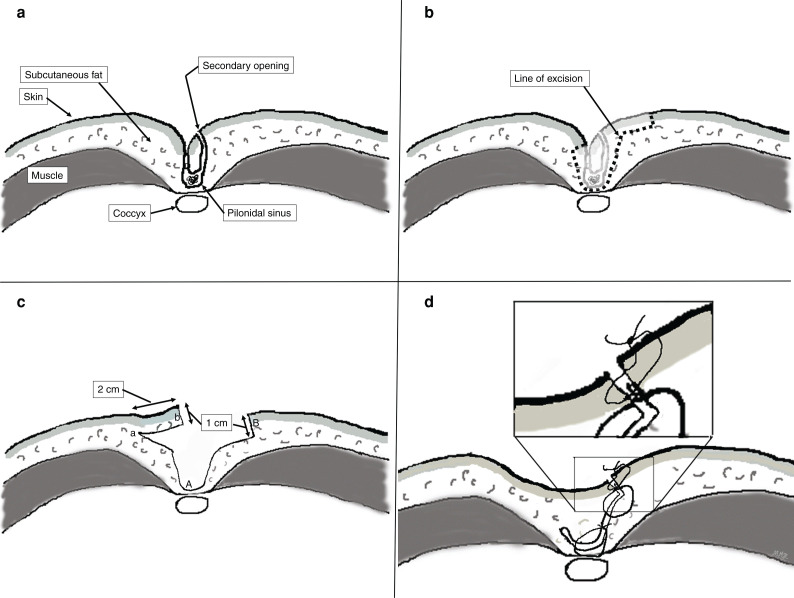
Relevant anatomy, line of excision, creation of flap and sutures in Karydakis flap surgery

All patients received antibiotic prophylaxis with a single dose of 1 g metronidazole and 160 mg sulfamethoxazole combined with 800 mg trimethoprim as an intravenous injection prior to surgery. One gram of paracetamol and a standard dose of a non-steroidal anti-inflammatory drug were given as premedication. All operations were performed under general anaesthesia. The skin was shaved in the wound area to promote wound healing both in the operating room and later during wound care if healing complications occurred. In both interventions, surgery started and ended with a 20 ml injection dose of a local anaesthetic subcutaneously in the wound area. The standard intervention arm involved excision of all tissue affected with pilonidal sinus disease with a symmetric elliptical incision centred in the midline and subcutaneous incisions performed lateral enough to allow tension-free midline closure and primary suture of the wound. A gauze roll sutured on top of the wound was used as a compression wound dressing. The experimental intervention arm was Karydakis flap surgery^9^, with minor modification. A symmetric elliptical excision was centred 2 cm lateral to the midline at the side with the most affected tissue or secondary fistula openings in the skin of the buttocks (*Fig. 1*). All affected pilonidal sinus disease tissue was removed with some normal tissue, enabling a lateral shift of the final closed wound. A 2-cm wide and 1-cm-thick skin flap was mobilized from the contralateral side to cover the skin defect and create a new natal cleft, with normal skin in the midline (*Fig. 2*). A modification of the original technique consisted of using a continuous 2-0 monofilament polydioxanone suture (PDS II) for the two deep layers of suture lines to secure the flap in its new position. A compression dressing made of a polyester fibre pad was placed on the closed wound.

The blue line indicates undermining of the flap, and the dotted black line is the centre axis of the skin excision positioned 2 cm lateral from the midline.

**a** Illustration of the pilonidal sinus with relevant anatomy in cross section. **b** Line of excision in Karydakis flap surgery illustrated in cross section. **c** Incision for flap creation in Karydakis flap surgery illustrated in cross section. **d** Cross section illustration of suture placement for securing the flap and wound closure in Karydakis flap surgery.

In both interventions, incisions were made using diathermy, and bleeding was controlled by diathermy coagulation. No dye was used to indicate the pilonidal sinus cavity or tracks and no drains were used.

All patients were discharged the same day with simple analgesia prescribed for 2 weeks and invited to follow-up visits until complete wound healing. Return visits were recorded by the case report forms for the study and the hospitals’ computerized patient record system. Routinely, the compression gauze roll or fibre pad was removed on day 3 or 4, and a thin wound dressing was applied. Skin sutures were removed on days 11 to 14 (not on weekends). Nurses responsible for follow-up of wound healing were instructed on standard wound care according to study protocol, case report forms detailing wound healing issues, and documenting when complete wound healing occurred. Impaired wound healing involving infections led to consultation with the attending surgeon for wound care instructions. An infected wound was sufficiently opened to drain any infected wound cavities, followed by washing out with saline. Subcutaneous wound cavities were either packed with Sorbact^®^ (ABIGO Medical, Askim, Sweden) or left open. Open wounds were covered with an absorbing pad. Wound dressings were changed daily, or more often if required, to keep the outer surface dry. Rinsing of infected open wounds at home with a shower twice daily was encouraged. Antibiotics were prescribed based on the surgeons’ judgement.

Specific surgeons were involved in each intervention arm, but no other adjustments to routine surgical practice were made. The surgeons involved at the two hospitals chose one of the two methods and performed only their selected procedure. Three surgeons chose to perform the excision with primary suture in the midline and another three performed the Karydakis flap surgery. All surgeons involved were specialists in general surgery and had more than 10 years of surgical experience.

A detailed illustrated manual (in Swedish) for both methods (Supplementary material) and a video of the Karydakis flap surgery (Video 1) were given to all involved surgeons, who were required to perform at least 10 operations of their choice according to the manual before the start of the study. For standardization purposes, two operations had to be performed together with the corresponding author.

### Outcomes

Co-primary outcomes were time to complete wound healing and recurrence rates. Wound healing was evaluated at the outpatient clinic at 1- to 2-week intervals until complete wound healing was documented. Complete wound healing was defined as a dry scar requiring no further care or dressing. Nurses responsible for postoperative wound care and registration were not involved in the surgery. The time elapsed from surgery to the day of suture removal was defined as the shortest possible wound healing time if no wound complications occurred within 30 days.

Recurrence was evaluated by a surgeon, who was not involved in the surgery, at the 3-, 12-, and 36-month follow-up visits, and with a telephone interview at the end of the study. In addition to the planned follow-up visits, all patients were instructed to contact the operating centre if any problems with the wound or scar occurred. The telephone interview was structured and conducted according to a specific data form by a surgeon or research nurse. Both were blinded regarding the intervention performed. If a wound opened within 30 days of surgery, it was considered a wound healing complication with delayed healing, affecting wound healing time. Recurrence of pilonidal sinus disease (31 days or later) was defined as a later opening of a healed wound, as well as any openings in the scar, nearby skin, or formation of a painful subcutaneous mass in the intergluteal area.

Secondary outcomes were postoperative wound complications that were categorized by two methods, open (or opened) postoperative wounds (yes or no), and separately with the Southampton Wound Assessment Scale^13^. Open postoperative wounds included a minor wound opening to total dehiscence, with or without purulent infection. Measures of postoperative open wound length, width, and depth were recorded, as well as details of wound care, at each visit. The largest measurements of the open wounds during follow-up were used for the report.

Additional secondary outcomes, according to the study protocol (health-related quality of life, time taken to return to normal physical activity after surgery, days spent on sick leave, healthcare costs and total costs), will be reported separately.

### Randomization and concealment mechanism

The randomization sequence was computer-generated using a random number technique and concealed. After informed consent was obtained, allocation to a study arm was provided online from a secure web-based registration and randomization application. Participants were randomly assigned to either the control or experimental arm with a 1:1 allocation. Permuted blocks were used, and randomization stratified by centre. The size of the permuted blocks varied by chance within predefined concealed limits. The permuted block sizes were not disclosed to the participating clinics. The entire process of registration, randomization, and allocation was completed on the study homepage, with coding performed by a system developer independent of the remainder of the study recruitment, conduct, and analysis. The centre-specific allocation sequences were stored on a secure server, accessible only by the above system developer at Information and Communication Technology Services and System Development, Umeå University, Sweden.

The original study protocol in Swedish can be downloaded from https://www.norrlandskirurgi.se/Randomisera/Blanketter/Psin/PSIN_Studieprotokoll_V8.pdf.

### Statistical analyses

Analyses were performed with the intention-to-treat principle. Median wound healing time and 95 per cent confidence interval (c. i.) was calculated with survival analysis, using time to event (complete healing) and allowing censoring (loss to follow-up). The log-rank test was used to compare wound healing time between the groups and the result was visualized with a Kaplan–Meier failure graph. Differences in proportions between groups were tested with Fisher’s two-sided exact test. Study groups were also compared with the Southampton Wound Assessment Scale, treating this variable on an ordinal scale, using the Mann–Whitney U test.

The power calculation was based on reported figures for recurrence: 2 per cent for Karydakis flap surgery^14,15^ and 10 per cent for excision and primary closure in the midline^10,16^. A requirement of 400 patients was estimated to achieve 90 per cent statistical power at a 5 per cent significance level (with an assumed 10 per cent loss to follow-up). According to the literature at study inception, no data existed on differences in wound healing time, providing no further information for a power calculation. An interim analysis was planned to take place after the first 100 operations. To balance for multiple testing, a Benjamini–Hochberg procedure with a 5 per cent false-discovery rate was used to determine statistical significance for the two co-primary endpoints in the study.

All tests were two-sided, and test results were considered significant if the *P* value was less than 0.05. Data analysis was performed in STATA (release 15; StataCorp, College Station, Texas, USA).

## Results

**Fig. 3:**
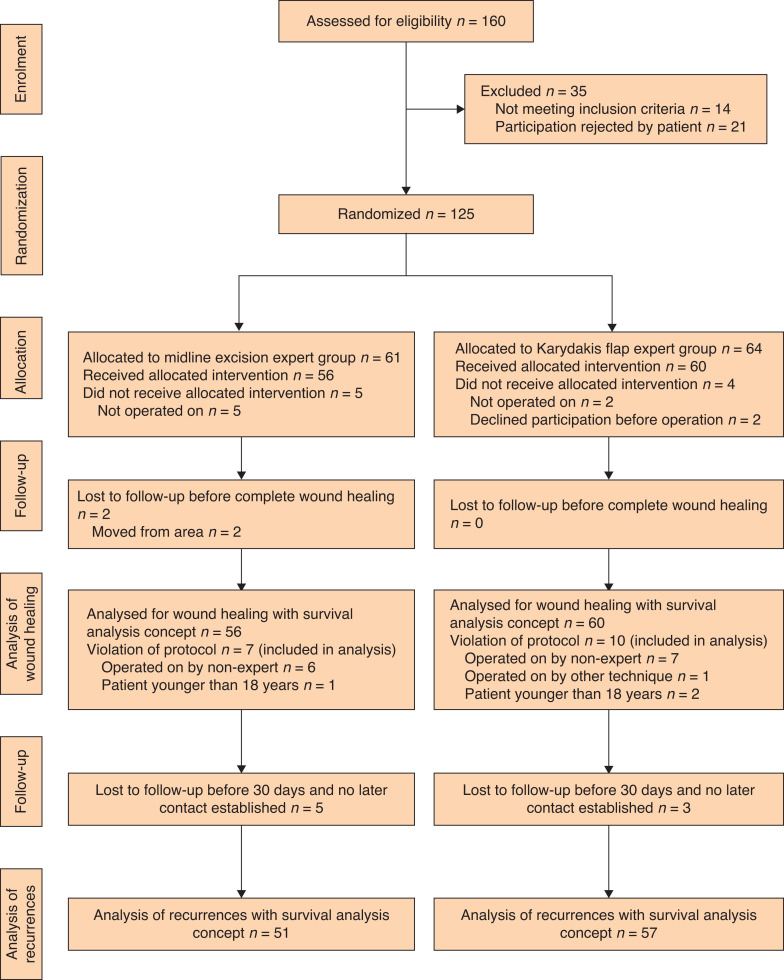
CONSORT diagram of study patients

The surgical department at Umeå University Hospital, in Umeå, Sweden, and the surgical department at Sunderby Hospital, Luleå, Sweden, participated in the study from September 2006 to March 2020 (first randomization to last long-term follow-up). An independent Professor of Surgery (Pär Nordin; Östersund Regional Hospital, Östersund, Sweden) recommended early termination of the study based on the results of a planned interim analysis of the first 100 patients recruited. The preliminary results suggested a significant difference in wound healing time between the groups. Guidance was provided by the Regional Ethical Review Board, which agreed to early termination of the study. Further inclusion of new patients was stopped in September 2015, when 125 patients had been enrolled and randomized. Of these, 116 could be analysed, with 56 and 60 patients in each group. Seventy-three patients were operated on at Sunderby hospital and 43 at Umeå University Hospital. The intention was to include consecutive cohorts at each hospital. However, 43 patients were treated by visiting surgeons, specialists, or residents not involved in the study and not assessed for eligibility in the inclusion period from September 2006 to September 2015. A CONSORT diagram of the study is presented in *Fig. 3*. Of the 116 patients, 103 were men. Patient characteristics are presented in *Table 1*. Median wound healing time was 51 days (95 per cent c.i. 34 to 66) for excision with primary suture in the midline and 14 (95 per cent c.i. 12 to 20) days for Karydakis flap surgery, calculated with the survival analysis concept. This difference was statistically significant (*P* < 0.001).

Karydakis flap, Karydakis flap surgery group; midline excision, radical excision and primary tension-free suture of the wound in the midline operation group.

**Table 1:** Patient characteristics

	Midline excision *n* = 56	Karydakis flap *n* = 60
Age (years)*	28 (23 to 29)	26.5 (23 to 30)
**M : F**	51/5	52/8
**ASA 1/2/3**	44/9/0	50/8/1
BMI (kg/m^2^)*	26 (25 to 27)	27 (26 to 29)
**BMI > 30**	9 (16)	13 (22)
**Current smoking (yes/no/missing)**	12/36/8	17/36/7
**One earlier operation for pilonidal sinus disease^†^**	3 of 52 (5.8)	5 of 58 (8.6)
**Earlier abscess**	26 of 53 (49)	24 of 55 (44)
Fistula length (mm)*	25 (20 to 35)	25 (16 to 30)
**Fistula length > 30 mm (yes/no/missing)**	19/32/5	19/37/4
**Secretion from skin opening when local pressure applied (yes/no/missing)**	31/23/2	26/33/1
**Skin opening communicating with pilonidal sinus (yes/no/missing; tested with thin probe)**	49/5/2	46/14/0
**Skin openings in midline (yes/no/missing)**	47/6/3	53/4/3
1–2	28 of 53	38 of 57
3 or more	19 of 53	15 of 57
**Skin openings lateral to midline (yes/no/missing)**	18/25/13	26/27/7
**Loose hair shafts in skin opening (yes/no/missing)**	20/34/2	18/42/0

Values in parentheses are percentages unless otherwise stated; *values are median (95% c.i.). ^†^One earlier operation for pilonidal sinus disease does not include earlier draining of an abscess. Karydakis flap, Karydakis flap surgery group; midline excision, radical excision and primary tension-free suture of the wound in the midline operation group.

**Fig. 4:**
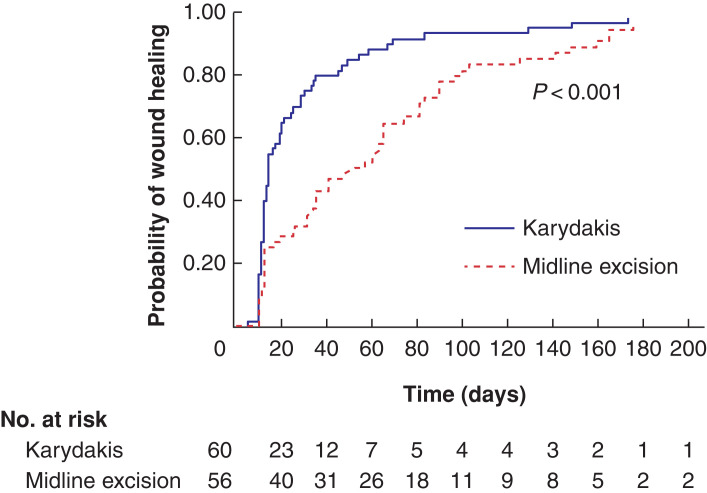
Kaplan–Meier graph illustrating time to complete wound healing for the two study groups

Wound healing time for the two different operations is illustrated in a Kaplan–Meier (failure) graph in *Fig. 4* and results are summarised in *Table 2*. Median operation time was 31.5 min (95 per cent c. i. 25 to 36) for excision and suture in the midline group, and 60 min (95% c.i. 50 to 69) for Karydakis flap surgery (*P* < 0.001).

Karydakis flap, Karydakis flap surgery group; midline excision, radical excision and primary tension-free suture of the wound in the midline operation group.

**Table 2:** Operative results

	Midline excision *n* = 56	Karydakis flap *n* = 60	*P*
Days to complete wound healing**	51 (34 to 66)	14 (12 to 20)	<0.001*
**Patients with open wound in the postoperative period (*n*)**	40	22	<0.001^‡^
Length of open wound (mm)**	31 (16 to 49)	25 (13 to 33)	0.543^†^
Width of open wound (mm)**	15 (6 to 20)	5 (3 to 10)	0.150^†^
Depth of open wound (mm)**	20 (12 to 30)	10 (3 to 20)	0.086^†^
**Healed wound at 3 months**	42/55	57/60	0.006^‡^
**Healed wound at 1 year**	54/55	59/60	1.00^‡^
Operating time (min)**	31.5 (25 to 36)	60 (50 to 69)	<0.001^†^
**Loose hair shafts in subcutaneous sinus (yes/no/missing)**	32/13/11	31/21/8	0.332^‡^
**Antibiotics in the postoperative period**	10	7	0.306^‡^
**Hospital stay (days on postoperative daycare ward)**	<0.5	<0.5	n.a.
**Recurrence (yes/no/missing)**	5/46/5	5/52/3	0.753*

**Values are median (95% c.i.). *Log-rank test. ^†^Mann-Whitney test. ^‡^Fisher’s exact test. Karydakis flap, Karydakis flap surgery group; midline excision, radical excision, and primary tension-free suture of the wound in the midline operation group; n.a., not analysed.

**Fig. 5:**
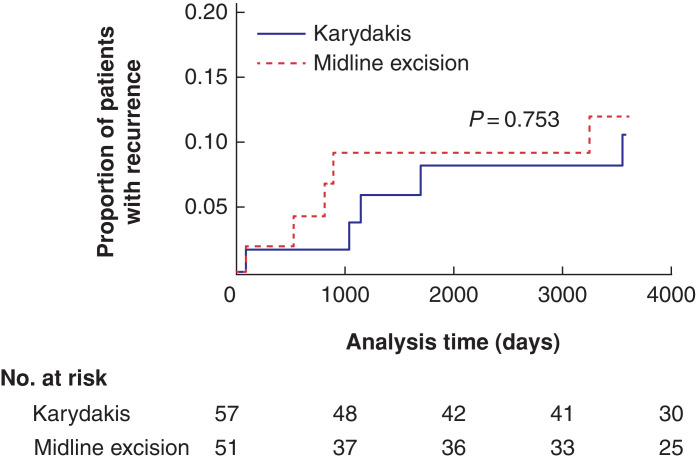
Kaplan–Meier graph illustrating the proportion of patients with recurrence registered by time

Median follow-up time was 3940.5 (range 11 to 5069) days. Successful follow-up as per protocol for a minimum of 3 years if no recurrence was achieved in 90 of 116 patients. There were 10 recurrences, of which five occurred in each group (*P* = 0.753). Recurrences were registered at postoperative day 88, 535, 803, 894, and 3245 for the excision and suture in the midline group and at postoperative day 91, 1033, 1142, 1688, and 3547 for the Karydakis flap group. The estimated frequency of recurrences for both groups by time is illustrated in *Fig. 5*. One recurrence occurred within 3 months in each group.

Karydakis flap, Karydakis flap surgery group; midline excision, radical excision and primary tension-free suture of the wound in the midline operation group.

Patients with open postoperative wounds were more common in the excision and suture in the midline group (40/56 *versus* 22/60; *P* < 0.001). Wound healing categorized according to the Southampton Wound Assessment Scale is presented in *Table 3*. There were more disturbances of wound healing in the excision and suture in the midline group than in the Karydakis flap surgery group (*P* < 0.001).

**Table 3:** Wound healing graded with the Southampton Wound Assessment Scale

Southampton Wound Assessment Scale		Midline excision *n* = 56	Karydakis flap *n* = 60
Description	Grade		
**Normal healing**	0	16 (29)	37 (62)
**Normal healing with mild bruising or haematoma**	I	2 (4)	1 (2)
**Erythema plus other signs of inflammation**	II	7 (12)	6 (10)
**Clear or haemoserous discharge**	III	13 (23)	11 (18)
**Pus**	IV	14 (25)	5 (8)
**Deep or severe wound infection with or without tissue breakdown; haematoma requiring aspiration**	V	4 (7)	0 (0)

Data are *n* (%). *P* = 0.002, Fisher’s exact test for a global difference between groups. *P* < 0.001, with the Mann-Whitney U test of operation groups when the Southampton Wound Assessment Scale was treated as an ordinal variable.

## Discussion

In this RCT, the Karydakis flap was superior in terms of shorter wound healing time and wound complications *versus* excision with primary tension-free suture in the midline. The study was terminated early on the basis of a planned interim analysis based on healing time. There was a statistically significant median difference of 5 weeks for the wounds to heal between the two studied patient groups. Recurrences may occur at any time after surgery and in the present study there were new recurrences registered up to 9 years after the operation, although there was no difference in the long-term recurrence rate between the two groups.

The wound healing time after Karydakis flap surgery in this study is in keeping with the literature^17,18^. Numerous observational studies of operations for pilonidal sinus disease have been undertaken, despite only one randomized study^12^ that compared the same techniques as the present study. In the previous randomized study, healing time was not an outcome and no significant differences in postoperative complications or recurrences were found^12^. A different definition of wound healing time was used in the present study than most other studies^6^, which may explain the present finding of a longer wound healing time in the excision and suture in the midline group. Most other studies have defined wound healing time as the period between surgery and removal of stitches for the sutured groups, regardless of what happens with the wound in the postoperative period. This does not adequately capture open wounds or those healing by secondary intention. The definition of time to complete wound healing (time from operation until there is a dry scar requiring no further special care or dressing) seems to be more appropriate for all techniques. This study did not show any significant difference in recurrence rate, in contrast to a previous study (2.5 *versus* 11.0 per cent), favouring the Karydakis flap operation, possibly due to shorter follow-up time (mean 30.4 months)^11^. In contrast to another randomized trial^12^, the present study revealed significantly more postoperative wound complications associated with a longer wound healing time in the excision and primary suture in midline group. Similar results have been found elsewhere^11,19,20^, raising questions about why midline wounds are associated with dehiscence or surgical site infections. Tension in the suture line is a possible cause^21,22^, but it has not been proven whether small excisions in the midline would produce more tension than large off-midline wounds. The theory of pressure changes in the subcutaneous tissue of the natal cleft when the patient stands up from a sitting position has been proposed as a primary cause of pilonidal sinus disease^23^. This mechanism might also result in postoperative midline wound complications.

Karydakis flap surgery is more complex than the excision and primary tension-free suture in the midline, clearly demonstrated by procedure time, which was approximately twice as long as the excision with primary suture in the midline technique. One of the negative aspects of Karydakis flap surgery is the long vertical scar it produces and a minimum wound length of 12 cm. In combination with the relatively large wound area under the flap, the authors consider the operation unsuitable for local anaesthesia alone, in contrast to many other less extensive techniques. The present follow-up time is long (6 to 13 years) and must be considered a strength, although there were some dropouts during the follow-up period. While the results from the present study can be generalized for a young male population with manifest pilonidal sinus disease, these outcomes may not be applicable in children or females.

There are limitations to this study. The study was terminated early, partly owing to slow recruitment, and also as a result of a planned interim analysis, which demonstrated a significant different in the primary endpoint. The study was only partly blinded, indicating that only the assessors of late recurrences were blinded to the type of operation performed. The assessors of early results (nurses at the outpatient clinic) were impossible to blind for type of operation but were not involved in the surgery or the analyses of the results. Another limitation was recognized regarding the planned follow-up by visits to the clinic 3, 12, and 36 months after surgery. This strategy largely failed, resulting in incomplete follow-up data. To compensate for this, a structured telephone interview was performed. The study did not include all consecutive patients, and some were not operated on by the preselected surgeons providing the standardized study treatment options, but they were analysed according to the intention-to-treat principle.

This was a pragmatic study in that no other adjustments to routine surgical practice were made, making the results easily applicable. The six surgeons in the study were experienced general surgeons but did not claim to be subspecialists in pilonidal sinus disease surgery, which may affect the generalizability of the results.

As the proportion of patients with complication-free postoperative wound healing was very low in both groups (29 per cent *versus* 62 per cent), the present study implies more research on wound healing and prevention of postoperative complications after surgery for pilonidal sinus disease is required.

## Supplementary Material

zrac007_Supplementary_Data

## Data Availability

The corresponding author can provide an anonymized patient data set from this trial to individual researchers upon request if they consent to publish data at a group-level only. The ethical approval and our interpretation of the General Data Protection Regulation in the European Union prohibit data publication at an individual level.
