# Supplementary material for: Excision and suture in the midline versus Karydakis flap surgery for pilonidal sinus: randomized clinical trial
Source: BJS Open. 2022 Mar 15;6(2):zrac007. doi: 10.1093/bjsopen/zrac007 (PMC8932511; doi:10.1093/bjsopen/zrac007)
Supplement: zrac007_Supplementary_Data [file zrac007_Supplementary_Data.zip › Supplementary_material.docx]

Operationsinstruktion för pilonidalsinus i PSIN studien

# Krav på operatör i PSIN studien

För att kunna ansvara för operationerna i endera expertgruppen krävs minst tio självständiga operationer med respektive operationsmetod varav minst två operationer är standardiseringsoperationer tillsammans med någon i studiegruppen.

# Antibiotikaprofylax

En dos antibiotikaprofylax med Metronidazol 1 g och Eusaprim 10 ml injiceras intravenöst vid operationsstart. Samma profylax för båda operationstekniker. Vid överkänslighet mot sulfa kan Eusaprim bytas mot en dos Zinacef 1,5 g intravenös injektion.

# Smärtprofylax

Patienten får paracetamol 1 g och diklofenak 50 mg tablett preoperativt.

# Narkos och lokalbedövning

Alla patienter opereras i generell anestesi eller spinal anestesi. Dessutom ges lokal anestesi för att säkra att patienten är relativt smärtfri i det omedelbara postoperativa förloppet. Använd en 50/50 blandning av Narop 7,5 mg/ml och Carbocain 10 mg/ml. Ge 20 ml av blandningen subcutant vid operationsstart och ytterligare 20 ml vid avslut. Maximal dos under en operation är 60 ml. Samma lokalbedövning används för båda operationstekniker.

# Kirurgisk teknik

**Inför steriltvätt och dukning på sal för båda operationstekniker**

Bukläge förbereds med kudde under höften och underben samt mjuk gelplatta under knäna (bild 1). Då patienten är placerad på operationsbordet rakas operationsområdet med elrakapparat som inte skadar huden (bild 2).

Gluteusmuskulaturen tejpas isär med drag mot operationsbordet på båda sidor (bild 3). Operationsområdet steriltvättas och dukas.

# Dissektion med diatermi i båda operationstyper

Vi rekommenderar gul knapp (cut) med 40W både för att skära och att koagulera mot pincett. Vill man ha mera skärande och mindre koagulation ökar man på effekten och vill man ha mera koagulation minskar man effekten till 20-30W. Effektläge 40W är en bra kompromiss som skär ganska torrt och det går att koagulera med pincett.

Blå kanpp (koag) rekommenderas inte pga att den automatjusterar upp spänningen till 16 000V genast det inte är bra kontakt. Fenomenet leder till att det slår ljusbåge över till närmaste vävnad med brännskador i hud som följd. Det slår också lätt gnista igenom dubbla handskar och det gör ont. Blå knapp är bara till för sprayfunktion.


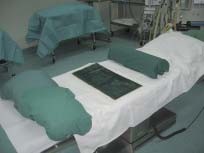


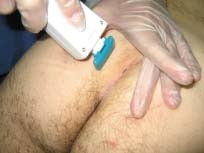

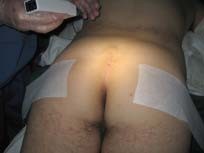


*Bild 1. Operationsbord förberett. Bild 2. Rakning av*

*operationsområde.*

*Bild 3. Tejpning av glutéer.*


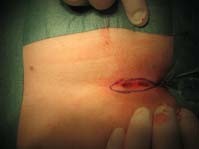

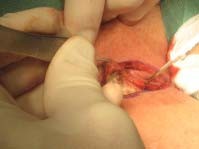

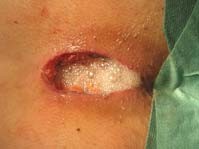


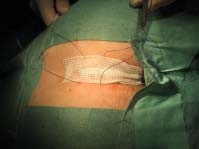
*Bild 4. Rita excisionen. Bild 5. Excision med diatermiskalpell. Bild 6. Sköljning med väteperoxid.*


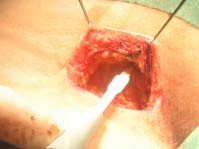

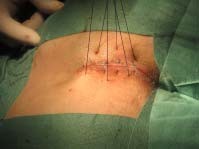


*Bild 7. Fria hudkanten. Bild 8. Hudsuturer och stödsuturer för kompressrulle.*

*Bild 9. Sårbandage.*

# Medellinjeoperation

I operation med oval excision i medellinjen medtas samtliga fistelöppningar. Beskrivningen som följer är stegvis och korrekt ordningsföljd. Hela operationsområdet ner till trochanter major skall rakas noggrant. Först ritas den tänkta incisionen ut på huden med steril märkpenna (Bild 4). Om det finns laterala utlöpare medtas dessa i excisionen, alternativt görs en kilexcision vinkelrät mot huvudincisionen. Därefter infiltreras 20 ml lokalbedövning i ett rombmönster kring den tilltänkta incisionen. All dissektion görs med diatermi (valfri utrustning ex epitom, nåldiatermi etc, inställningar se ovan). Sträva efter att inte få hål på fistelsystemet och säkerställ att all inflammerad vävnad tas bort. När preparatet är borttaget blodstillas såret noggrannt. Såret spolas sedan med väteperoxid (Bild 6) och efter detta plockas alla gamla kompresser bort och endast nya används. Kanterna undermineras sedan 1-2 centimeter lateralt för att undvika spänning i såret (Bild 7). Dissektionsplanet för undermineringen bör ligga 1 cm djupt om hudplanet. Noggrann blodstillning igen, med diatermi. Det sätts 2-4 grova hållsuturer med 2:an Ethilon (eller motsvarande ex PDS 1:an ) som går ca 1cm från hudkanten, genom botten av sårhålan. Huden sutureras med Ethilon 3-0 (eller motsvarande), stående madrassuturer. Därefter knyts hållsuturerna över kompressrullen. Undvik att dra åt suturerna för hårt. Infiltrera ytterligare 20 ml lokalbedövning runt såret. Ett tryckförband anläggs med 3 stycken hoprullade 20x30 cm absorberande kompresser, en på varje sida kompressrullen och en ovanpå. Dessa fixeras med Mefix (eller motsvarande) som dras hela vägen ner till tro- chanter major. Var noggrann med fixeringen kring anus.

Operationsmetoden är beskriven av Aldean et al (Colorectal Dis 2005 Jan;7(1):81-5 ) samt Odensten et al (Abstrakt P89 kirurgveckan 2006).


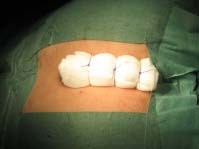

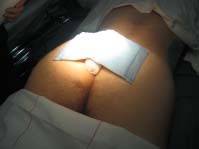

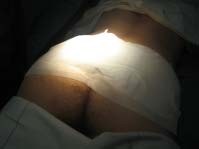


*Bild 10. Kompressionsrulle fixerad. Bild 11. Yttre sårbandage. Bild 12. Kompressionsförband överst.*


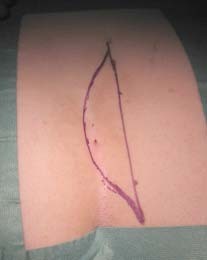

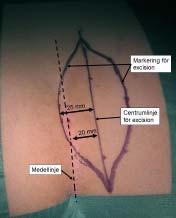

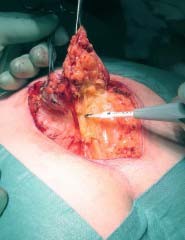


*Bild 13. Rita för excisionen medialt med liten marginal och symmetri till den laterala linjen.*

# Karydakis operation

*Bild 14. Rita för hela excisionen. Bild 15. Excision med diatermiskalpell.*

Snittet planeras och ritas enligt följande. Medellinjen markeras med några punkter varefter man märker ut några punkter 2 cm lateralt till den sida som är mera angripen av sjukdomen och en linje parallell med medellinjen ritas 2 cm lateralt från medellinjen. Den laterala linjen bildar nu en centrumaxel för en ovalär excision som tar med hela fistelsystemet. Man ritar snittets mediala sida från den laterala linjen till medellinjen och över minimalt på andra sidan med liten marginal till fistelsystemet och tillbaka till den laterala linjen (bild 13). Excisionen ritas symmetriskt på lateralsidan om den laterala linjen med att mäta avstånden från linjen till mediala markeringen och rita på samma avstånd från linjen på lateralsidan (bild 14). Excisionen blir då minst fyra cm bred på bredaste stället men ofta 4,5-5,5 cm beroende på att excisionen ofta bör gå över på andra sidan några mm för att få med hela fistel- systemet. Längden på excisionen bör planeras ungefär tre gånger bredden för att senare få en förslutning utan ”öronbildning” i spetsen på såret. Då snittet är ritat lokalbedövar man med 20 ml subkutant fördelat runt kanterna på den planerade excisionen.

Man gör en bikonvex incision enligt den ritade markeringen, och som medtar fistelöppningarna. Excisionen place- ras på den sida som är mest angripen eller har sekundäröppning. Det kommer en hel del frisk hud med i excisionen på lateralsidan om linjen (bild 15). Excisionen görs i helhet med diatermiskalpell medan god hemostas bevaras och blödande kärl koaguleras. Lateralt görs snittet 1 cm djupt rakt in i fettet varefter man vinklar in mot mitten och under fistelsystemet (bilder 16-17). I de mediala delarna av såret skall excisionen gå runt och under fistelgångar med liten men klar marginal och man kommer ofta ned till fascia som täcker sacrum i medellinjen.

Noggrann blodstillning görs med diatermi och såret spolas med väteperoxid.

En medial lambå skapas med snitt två cm in i fettet på medialsidan från kanten (på en centimeters djup i fettet) (bilder 18-20). I detta skede bör tejpdraget mellan operationsbord och gluteus på båda sidor avlägsnas så att hud och subcutis blir mera rörligt i operationsområdet. Lambån förflyttas lateralt över medellinjen och sutureras ner mot mitten av sårhålan med fortlöpande resorberbar monofil sutur (2-0 PDS, Maxon eller Biosyn) i två sub- kutana lager (bild 21-23). Det djupare suturlagret fixerar djupaste fickan i fettet under lambån ner mot medellinjen


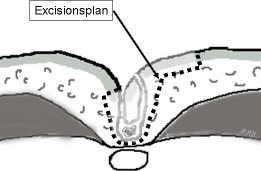


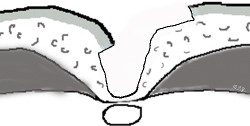

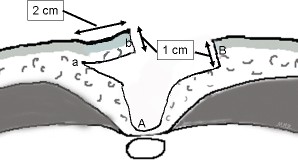


*Bild 16. Excisionsplan i tvärsnitt. Bild 17. Sår i tvärsnitt. Bild 18. Sår i tvärsnitt med mobiliserad*

*lambå som är 1 cm tjock.*


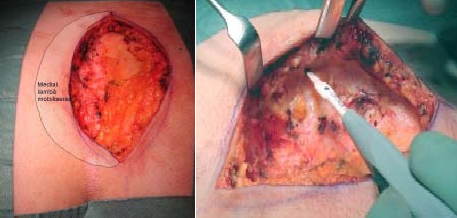

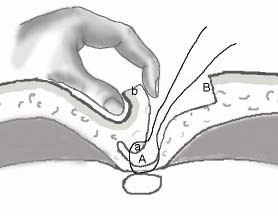


*Bild 19. Storlek av lambå medialt.*

*Bild 20. Lambån mobiliseras 2 cm in i fettet. Bild 21. Botten av mobiliseringen*

*sutureras ner mot sacrum.*

mot fascian över sacrum och då kommer lambån två cm över på andra sidan. I originalbeskrivningen används enskilda suturer för fixeringen av den mediala lambån och det kan vara tekniskt något enklare än den fortlöpande suturen som vi använder. Sutureringen underlättas betydligt om en assistent kränger upp kanten av lambån och trycker den samtidigt mot sin nya position. Observera att huden inte mobiliseras på lateralsidan av excisionen.

Hudkanterna kommer ändå att gå ihop efter att lambån har fixerats i sin nya position och den icke mobiliserade laterala hudkanten bildar ett nödvändigt motdrag så att hudsåret kommer att ligga 2 cm lateralt om medellinjen. Om hudexcisionen lateralt har gjorts mindre (i den friska huden) eller om den laterala hudkanten mobiliseras så kommer det nödvändiga motdraget att saknas och såret hamnar i medellinjen vilket inte är önskvärt i denna tek- nik. Hudkanterna sutureras med Ethilon 4-0 med ½ madrassuturer med instick från laterala hudkanten så att mediala kanten i lambån inte får suturhål upp till hudytan. Det förslutna såret bör ligga 15-20 mm lateralt om medellinjen som nu täcks av lambån och frisk hud (bild 24). Man bör behandla huden i lambån varsamt under operationen och man vill undvika att penetrera huden i lambån med varken suturer eller nålstick. Sår, skador och hål i lambån kan underlätta för lösa hår att penetrera genom huden och därigenom öka risken för recidiv.

Tunt sårförband läggs underst (bild 25) och ett tjockt tryckförband av polstervadd (8x8x12 cm) fixeras med Mefix tejp över hela operationssåret (bilder 26-29). Vi använder inte sårdrän utan tror att tryckförbandet och noggrann hemostas under operationen förhindrar både bildning av hematom och serom.

Ett specialfall är när en fistelöppning ligger anusnära, då måste centrumlinjen i den ovalära excisionen vika av mot medellinjen. Ett annat specialfall, ännu ovanligare är när man har sekundäröppningar på båda sidor om crena ani. Då lägger man centrumlinjen av ovalära excisionen på den mest angripna sidan och gör en kilexcision av den an- dra fistelöppningen med basen mot medellinjen.


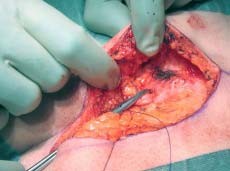

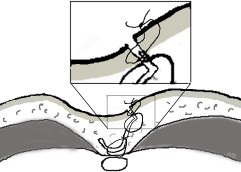

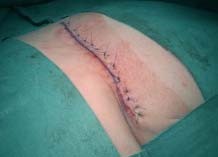


*Bild 22. Botten av mobiliseringen sutureras ner mot sacrum.*

*Bild 23. Lambåns subkutana lager sutureras i två skikt mot andra sidan.*

*Bild 24. Huden sutureras med ½-madrass suturer från laterala sidan.*


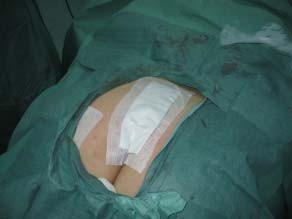

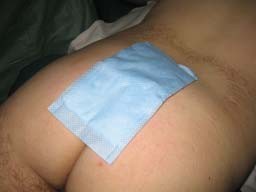


*Bild 25. Tunnt sårbandage läggs underst. Bild 26. Absorptionsdyna i nästa lager..*


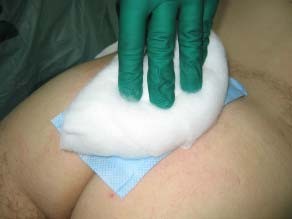

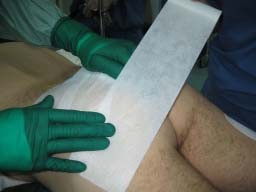


*Bild 27. Kompressionsförband tillverkas av polstervadd..*

*Bild 28. Kompressionsförbandet fästes med tejp.*


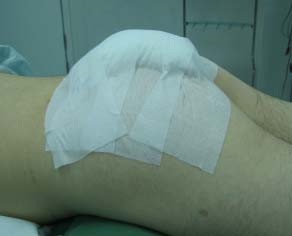


*Bild 29. Färdigt kompressionsförband som skall sitta 3-5 dagar.*

# Postoperativ vård

Patienten förses med lämpligt peroralt analgetika postoperativt. Återbesök sker efter 3-5 dagar på mot- tagningen då det stora tryckförbandet avlägsnas och det inre tunna sårförbandet ersätts efter inspektion av såret. Suturtagning efter 10-12 dagar och eventuellt ytterligare kontroll om några tveksamheter i sårläkningen uppstår. Vi rekommenderar att patenten avstår från full sportaktivitet ytterligare cirka 2 veckor.

Om läkningsproblem är vi aktiva med skrapning med slev av sårhåla i gelanestesi och spolning med vä- teperoxid och täta kontroller på kirurgmottagningen en gång per vecka.
